# Survival of biological therapeutics in psoriasis: retrospective analysis of 3-years data in a Turkish registry, PSORTAKSIS

**DOI:** 10.3906/sag-2104-339

**Published:** 2021-08-07

**Authors:** Kemal ÖZYURT, Gökmen ZARARSIZ, Ragıp ERTAŞ, Ahu CEPHE, Ömer KUTLU, Ömer Faruk ELMAS, Muhammed Reşat AKKUŞ, Fatma Nur KUTLU, Mustafa ATASOY

**Affiliations:** 1 Department of Dermatology and Venereology, Medicine Faculty, Ahi Evran University, Kırşehir, Turkey; 2 ERFARMA, Personalized Medicine and Advanced Analytics Research Group, Erciyes University, Kayseri, Turkey; 3 Department of Biostatistics, Faculty of Medicine, Erciyes University Kayseri, Turkey; 4 Department of Dermatology and Venereology, Kayseri City Hospital, Health Science University, Kayseri, Turkey; 5 Department of Dermatology and Venereology, School of Medicine, Tokat Gaziosmanpaşa University, Tokat, Turkey; 6 Department of Dermatology and Venereology, School of Medicine, Kırıkkale University, Kırıkkale, Turkey

**Keywords:** Psoriasis, drug survival, anti-TNF, ustekinumab, secukinumab, psoriasis registry

## Abstract

**Background/aim:**

PSORTAKSIS is a psoriasis registry, which is used for follow-up of patients in Kayseri City Education and Research Hospital, Dermatology Clinic since 2016 in Turkey. PSORTAKSIS includes demographic data, follow-up clinical findings, laboratory output, and treatment information of patients. Here, drug survivals of biologic therapeutics (BT) according to three-year data of PSORTAKSIS will be presented.

**Materials and methods:**

Drug survival of BT in PSORTAKSIS was analyzed from 2016 to March 2019.

**Results:**

158 patients (111 of them BT-naive) with psoriasis under BT were enrolled in the current study. Drug survival analysis of patients with ongoing BT (158 treatment periods) revealed mean survival time as 15.49 months for ustekinumab, 15.37 months for adalimumab, 14.00 months for etanercept, 5 months for infliximab, and 4.59 months for secukinumab. The differences between drug survivals of BT were statistically significant (log-rank test, χ^2^ = 79.915, *p* < 0.0001).

Age of onset was found to be the only independent risk factor of drug survival according to regression analysis (p = 0.029).

**Conclusion:**

As a conclusion, drug survival of UST was significantly higher than that of TNF-alpha inhibitors and SEC in the treatment of psoriasis. This study revealed that among predictors, age at disease onset may influence drug survival.

## 1. Introduction

WHO Global report on psoriasis regarding 67th World Health Assembly in 2014, was aimed to increase awareness of psoriasis, which is a common chronic skin disease that, due to frequent accompanying comorbidities, directly affects overall health and life quality of patients^1^. Psoriatic arthritis (PsA), inflammatory bowel disease, myocardial infarcts, congestive heart failure, psychiatric diseases, multiple sclerosis, viral hepatitis, and latent tuberculosis are some of the comorbid conditions that may be associated with the disease or side effect of prolonged treatments in psoriasis [1]. Expectedly, targets of treatments should include not only improving the discomfort and distress of patients caused by cutaneous lesions but also preventing and treating systemic effects and comorbidities.

Psoriasis has numerous choices of treatments including topical (TPC) therapeutics, phototherapy (PHT), and systemic drugs such as methotrexate (MTX), acitretin (ACN), cyclosporin (CYC)) and biologic therapeutics (BT). BT are particularly affected by targeting various genetic or immune mediators act in pathophysiology, like blocking the TNF-α, interleukins, which mainly cause inflammation or other manifestations of psoriasis [2]. BT have been used more frequently in recent years, and each has its unique features and limitations. Deciding the appropriate and realistic modality for both induction and maintenance phases of treatment is based on clinical, personal, and socioeconomic factors.

Researches based on patient registries and real-life experiences, and clinical trials provide safety and efficacy data to select the right choice among BT [3]. Patient registries and medical databases are increasingly being used to maintain medication compliance and persistence in a various disease. The reason of this effort is to gain more detailed healthcare data and a realistic economic picture of this issue [4].

Recently, drug survival is accepted as the duration of a specific therapy that persists beneficial for a patient from initial to discontinuation of treatment, indicating overall treatment success and compliance, also, reveals useful data on drug efficacy and safety and as well as patient satisfaction [5, 6]. Benner et al. stated that the drug survival rate, a measured probability to discontinue the drug, is a simple and clinically relevant measure of the treatment outcome [7]. Although some of its limitations are emphasized, drug survival is a useful tool, investigating BT efficacy on psoriasis in clinical settings. BT, which are remarked with prolonged drug survival, are accepted as more efficient and safer. The most important factor to being preferred is its availability based on clustered medical data of real-life experiences or registries [8,9].

The objectives of this study were to determine the drug survival of BT in psoriasis treatment and to compare drug survival of each biologic, also to assess the impact of additional factors: age, sex, onset age of disease, family history of psoriasis, BT-Naive status, body mass index (kg/m2), accompanying systemic disease, and PsA.

## 2. Materials and methods

The current study is conducted in Chronic Skin Diseases Unit (CSDU) of Dermatology Clinic, Kayseri City Training and Research Hospital (KCH) in March 2019. Ethical approval for this retrospective study was gained from Local Ethical Committee (20.03.2019-2019/228). CSDU has been established for the management of frequent chronic skin diseases, including psoriasis, chronic urticaria, and Behçet’s Disease since 2015 [10]. PSORTAKSIS (abbreviation of “Psoriasis Takip Sistemi” in Turkish) registry was developed by KCH Dermatology Clinic and embedded in Hospital Knowledge Management System software. PORTAKSIS has been used for patients with psoriasis since 2016 [4]. In PSORTAKSIS, electronic health records of patients, including demographics, body mass index (BMI), overall personal medical history, family psoriasis question, accompanying diseases, previous and current treatments, significant reports of patients about drug usages (adverse reactions, etc.), progress of disease and response to treatments, clinical type of psoriasis, nail and artrit involvements are systematically tracked.

This study is aimed to investigate drug survival of BT, which were included in PSORTAKSIS, Ustekinumab (UST), Adalimumab (ADA), Etanercept (ETN), Infliximab (INX) and Secukinumab (SEC), from 2016 to March 2019. Drug survival is accepted as the time passed from the initiation of the therapy to the discontinuation of the drug, in terms months [6]. Duration time of BT periods were obtained from PSORTAKSIS, and median of drug survival for each treatment period was calculated.

The treatment periods are determined as the duration between the initiation of BT and the time of discontinuation. Switching to another biologic process is also accepted as discontinuation. Treatment periods are accepted to start when at least one dose of BT included to the study. Any treatment period less than 1-month or with missing data about continuation of BT was excluded. Discontinuations of BT were due to switching, loss of efficacy, patient decision, or interruption in follow-up of patient with unknown cause. A total of 158 patients enrolled in the study with ongoing BT (PoBT). PoBT who did not exposed BT before were accepted as BT-Naive patients. Medical data and 62 previous BT treatment periods of 47 BT-nonNaive patients were explored. Twenty-four of them excluded from the study (18 were <1month period, 6 had missing data) and 38 treatment periods included. In this manner, 196 total treatment periods and 158 PoBT treatment periods were analyzed for drug survival, severally.

Also, age, sex, onset age of disease (<=40 years / >40 years), family history of psoriasis, accompanying systemic disease, previous usages of systemic drugs, body mass index (kg/m2), having psoriatic arthritis (PsA), and knowledge of BT-Naive status of PoBT were investigated as to be predictor factor for drug survival.

Histogram, q-q plots, and Shapiro–Wilk’s test were applied to assess the data normality. Pearson chi-square analysis was used for categorical comparisons. Bonferroni adjusted z test was conducted for multiple comparison analysis. Kaplan–Meier plots were generated, and Log-rank test was performed to compare the survival probabilities of total and PoBT treatment periods of ongoing BT survival. To identify the risk factors of drug survival, univariate and multiple Cox regression models were built. Significant variables at p < 0.25 level were included into multiple model, and backward elimination was used to detect the independent risk factors using likelihood ratio statistic. Hazard ratios are calculated with 95% confidence intervals. All analyses were conducted using R 3.5.0 (http://www.r-project.org) and TURCOSA (Turcosa Analytics Ltd. Co. Turkey, http://www.turcosa.com.tr) statistical software. A p value less than 5% was considered as statistically significant.

## 3. Results

Demographic data of PoBT involved to the study is summarized in Table 1. Mean age of 158 PoBT was 46.61±12.19. Fifty patients’ ages were <=40 and 108 of them were 40>. Male patients were 85 (53.8%) and female were 73 (46.2%). Onset ages of disease were <=40 in 81% of patients, in the rest 19.0% were>40. Family history of psoriasis observed in 28.5%. PsA was in 27.8 %, psoriatic nail involvement was in 28.5 %, psoriasis associated pruritus was in 68.4 % of PoBT. Mean BMI of PoBT was 28.79±5.53 kg/m2. Accompanying diseases were seen in 52 (32.9%) patients (Table 1). Twenty-one unique systemic diseases were 73 times seen in 52 patients. The frequency of accompanying diseases was showed in Table 2.

Table 3. showed previous non-BT of patients; 156 (98.7%) patients had TPC, 70 (44.3%) patients PHT and systemic therapies, 119 (75.3%) patients had MTX, 104 (65.8%) patients had ACN, and 61 (38.6%) patients had CYC.

Ongoing BT consisted of 78 patients UST, 49 patients ADA, 7 patients ETN and INX, and 17 patients SEC. A total of 158 treatment periods were defined among PoBT. 62 previous treatment periods (11 patients UST, 26 patients ADA, 15 patients ETN, 7 patients INX and 3 patients SEC) added, and, in total, 220 treatment periods were defined. As the exclusion criteria explained in Material and Methods section, 24 of them (UST:5, ADA:11, ETN:5, INX:3 and no SEC treatment) excluded. Excluding reasons were, less than 1-month duration in 18 and with missing data in 6 treatment periods Finally, included treatment periods were 196 (Table 4).

**Figure 1:**
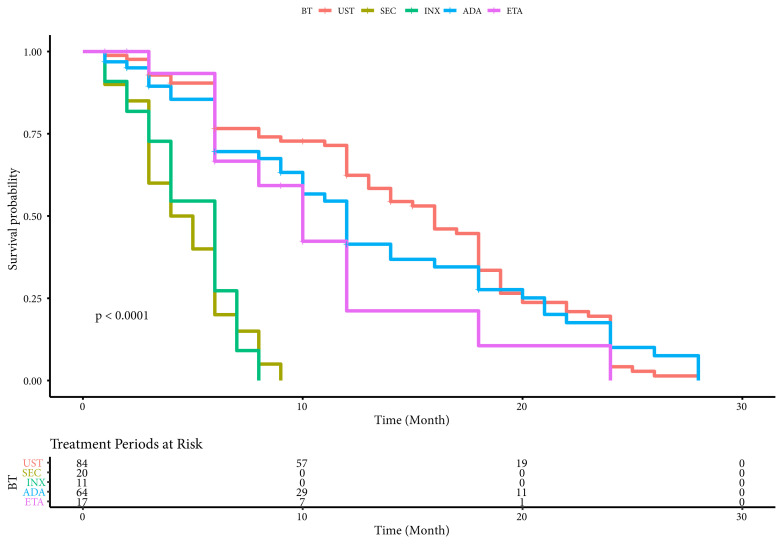
The differences between drug survivals of overall biological agents according to Kaplan–Meier graph.

Survival of included total 196 treatment periods of BT were demonstrated in Table 5. The mean survival time is 14.91 months for UST, 13.30 months for ADL, 11.07 months for ETN, 4.91 months for INX and 4.65 months for SEC (Table 5). The differences between drug survivals of BT were statistically significant according to Kaplan–Meier graph (log-rank test, χ2 = 80.688, p< 0.0001). Drug survival of ustekinumab was significantly higher than that of TNF-alpha inhibitors and SEC (Figure 1).

**Figure 2:**
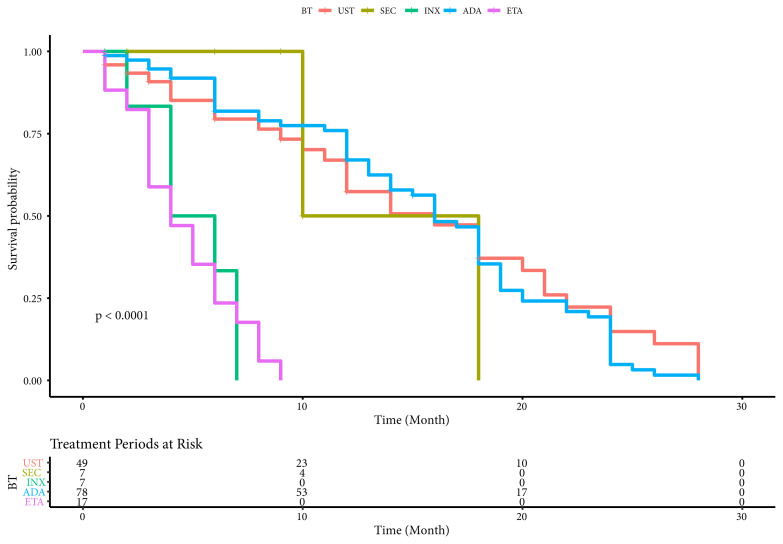
The differences between drug survivals of patients ongoing biological agents according to Kaplan–Meier graph.

Also, survival of 158 treatment periods of PoBT were showed Table 6. The mean survival time was 15.49 months for UST, 15.37 months for ADA, 14.00 months for ETN, 5 months for INX, and 4.59 months for SEC (Table 6). The differences between drug survivals of BT were statistically significant according to Kaplan–Meier graph (log-rank test, χ2 = 79.915, p < 0.0001) (Figure 2). Univariate and multiple Cox regression analysis were conducted in order to identify the risk factors of BT survival. After backward elimination, age of onset was found to be the only independent risk factor of drug survival. Patients with age > 40 had 1.71-fold higher risk of drug survival as compared to the patients with age <= 40. Among predictors, onset age of disease influenced drug survival significantly (Table 7).

## 4. Discussion

Although strong evidences revealed genetic, immune, and environmental factors in pathogenesis of psoriasis, it is not clear exactly how they interact with each other and which one is more effective. Being one of the prominent findings, history of psoriasis in relatives were reported 30%–50% of patients [11]. Similarly, this study determined 28.5 % of family history.

Hajiebrahimi et al. just reported that chronic inflammation is a common factor among psoriasis and its comorbidities. It was stated that severity of disease and accompanying comorbidities were seen more frequently over the years after the onset of psoriasis [12]. PsA, Crohn’s disease, diabetes mellitus, arterial hypertension, liver steatosis, chronic renal failure, psychiatric disorders, and malignancies are related to chronic inflammation in psoriasis [13]. Current study showed that 32.9% of associated diseases are arterial hypertension, diabetes mellitus, asthma, psychiatric disorders, etc. Patients with mean BMI of 28.79±5.53 kg/m^2^ were accepted as overweight. Onset ages of psoriasis were <=40 in 81% of patients. This may be supporting that longer duration of disease contributes with severity and accompanied comorbidities. PsA is seen in 5%–30% of patients, being a chronic inflammatory disease [14]. PsA were observed in 27.8 % of patients included to this study. Nail involvement was in 28.5% of the patients. It was demonstrated that earlier onset and familial psoriasis is associated with increased severity of skin and nail disease [15]. Patients ongoing with BT were included to this study and previous non-BT, systemic therapies were prevalent like 75.3% MTX, 65.8%, can, and 36.6% CYC (Table 3). Also, 27.7 % used at least one or more BT before the ongoing BT (Table 4). These findings showed that thepatients enrolled in this study were with high severity of psoriasis.

Treatment of patients included in PSORTAKSIS were treated according to the Turkish Guideline for the Treatment of Psoriasis-2016 [16]. BT were indicated in cases of moderate to severe psoriasis with rapid deterioration of clinic status, involvement of visible areas or causing functional insufficiency, severe erythrodermic or generalized pustular psoriasis. The patients who are unresponsive or develop contraindications/side effects to conventional systemic therapeutics are accepted as BT indication regarding their worsening clinic.

At the end of induction phase and assessments of patients during maintenance phase, PASI was used for determining severity of the disease. Improving 75% of initial PASI (PASI 75) was accepted as success, and the treatment continued. If the PASIs are between PASI 50-PASI 75, DLQI scores were considered, and if DLQI >5, it is accepted as a failure for current therapy, otherwise continued [16].

It is worthwhile to mention here that all five BT can be used under the same insurance drug payment terms in Turkey; the choice of BT depended mostly on dermatologists’ options. PSORTAKSIS allows detailed investigation of various treatment periods of psoriasis included since 2016 (Table 4). By exploring data of 158 patients included in the registry until May 2019, 196 treatment periods of BT were defined, and the median survival time was 16 months for UST, 12 months for ADA, 10 months for ETN, 6 months for INX and 4 months for SEC. UST was determined with significantly higher drug survival than that of TNF-alpha inhibitors and SEC (p < 0.0001) (Figure 1). Similar reports were published exhibiting higher drug survivals of UST and ADA over ETN [17–21]. Also, superior drug survival of UST has been emphasized comparing with TNF-α blockers in large registries and studies [22–24].

In consistency with the current study, a published report based on DERMBIO registry revealed that UST was associated with the highest drug survival, and SEC was found lowest (also ADA and INX included to the study). Authors of the study took attention to limitation for the lower number of SEC treatment periods. They remarked that asserting exact propositions should be cautious about SEC, regarding the presented data [25]. ADA, UST, INX, and ETN have been used for more than three years in Turkey. However, SEC has been used since June 2018. The longest treatment period of SEC was 9 months in PSORTAKSIS. Therefore, in this study, the drug survival of SEC therapy may be statistically not comparable with other BT. This was the limitation of this study.

Richter et al. stated that UST and ADA were marked by longer drug survival compared to ETN. They found no significant difference in drug efficacy as determined by PASI. This was an example to point out the bias of drug survival [26]. Carcia-Doval et al. claimed that drug persistence should be as a proxy convenient use of safety or effectiveness because of outcomes with an unequivocal meaning [27]. They notified that many challenging tasks are present to choose the true drug and manage psoriasis patient. Exploring survival of BT for psoriasis suggest efficacy of drugs in an identified time of real-life experience or registry. The maintenance of a prolonged treatment is accepted as its efficacy and is safety proven by physicians and, as well as patients’ compliance and benefits. Loss of follow-up caused by financial matters, problems of insurance, change of province, death or with other factors that cannot be known or interpreted by physicians, are disadvantages for determining factual drug survival [17]. However, detailed and comprehensive registries, potentially, permit drug survival of prolonged specific treatments that is invalid in randomized clinical trials composed strict clinical procedures and standardized evaluations.

In a study, the probability of discontinuation of BT was higher with PsA presence and BT-nonNaive. It was showed that only arthritis retained its statistical significance, and previous usage of BT was not independently significant in multivariate model [17]. Jacobi et. al. concluded that patients with metabolic syndrome demonstrate lower adherence to BT [28]. Another study showed that having one of comorbidities was a determinant of decreased ADA drug survival related to ineffectiveness. Also, researchers stated that men showed better drug survival as in other drug survival studies in psoriatic disease [21]. Shalom et. al. found UST with higher drug survival rate among ADA, ETN, and INX. Also, biologic naivety, metabolic syndrome, and male sex were observed positive predictors for drug survival. They enounced that presence of significant comorbidities such as diabetes mellitus positively effect patient awareness and increases BT compliance [29]. Current study searched the impact of probable predictors (Table 6). As it is mentioned above, 32.9 % presence of systemic disease association, overweight, earlier onset of disease, relatively higher arthritic, and nail involvements and family history all strengthen the evidences for higher disease severity of patients enrolled in the study. Regression analysis revealed that, among predictors, earlier onset age of disease influenced drug survival significantly (Table 7). Similarly, the results of the study of Shalom et. al may clearly conclude that the early onset and higher severity of diseases improve drug survival [29].

In conclusion, drug survival of UST was significantly higher than that of TNF-alpha inhibitors and SEC in treatment of psoriasis. Current study exposed that earlier onset age of disease is clearly associated with longer duration, more severe and frequent comorbidities, and it may be a positive predictor for drug survival in BT.

## Figures and Tables

**Table 1:** Descriptive statistics of demographic and medical data.

Variable	Biologic Therapy (*n* = 158)
**Age (years)**	46.61 ± 12.19
**Age**	
**<=40**	50 (31.6)
**>40**	108 (68.4)
**Sex**	
**Male**	85 (53.8)
**Female**	73 (46.2)
**Onset age of disease**	
**<=40**	128 (81.0)
**>40**	30 (19.0)
**Family history of psoriasis**	45 (28.5)
**Accompanying disease**	52 (32.9)
**Body mass index (kg/m** **^2^** **)**	28.79 ± 5.53
**Psoriatic arthritis**	44 (27.8)
**Nail involvement**	45 (28.5)
**Pruritus**	108 (68.4)

Values are expressed as *n* (%), mean±SD or median (1st–3rd quartiles).

**Table 2:** The distribution of accompanying diseases.

Accompanying diseases	*n* (%)
**Arterial hypertension**	18 (24.7)
**Diabetes mellitus**	16 (21.9)
**Asthma**	6 (8.2)
**Depression**	5 (6.8)
**Rheumatoid arthritis**	4 (5.5)
**Thyroid Disease**	3 (4.1)
**Ankylosing spondylitis**	3 (4.1)
**Gut Disease**	2 (2.7)
**Coronary arterial disease**	2 (2.7)
**Chronic renal failure**	2 (2.7)
**Chronic obstructive lung disease**	2 (2.7)
**Hepatitis B Disease**	2 (2.7)
**Panic Disorder**	1 (1.4)
**Fibromyalgia**	1 (1.4)
**Migraine**	1 (1.4)
**Chronic urticaria**	1 (1.4)
**Hypercholesterolemia**	1 (1.4)
**Congestive heart failure**	1 (1.4)
**Bipolar affective disease**	1 (1.4)
**Anxiety disorder**	1 (1.4)

**Table 3:** Previous non-biologic therapies.

Therapies	*n* (%)
**MTX**	119 (75.3)
**ACN**	104 (65.8)
**CYC**	61 (38.6)
**TPC**	156 (98.7)
**PHT**	70 (44.3)

Topical (TPC), Phototherapy (PHT), Methotrexate (MTX), Acitretin (ACN), Cyclosporin (CYC).

**Table 4:** The comparison of therapeutics among BT groups.

Variables	BT					Total (*n*=220)	*p value*
	UST (*n* = 89)	ADA (*n* = 75)	ETN (*n* = 22)	INX (*n* = 14)	SEC (*n* = 20)		
**TP (excluded/included)**							
**Excluded TP**	5 (5.6)**^a^**	11 (14.7)**^ab^**	5 (22.7)**^b^**	3 (21.4)**^b^**	0 (0.0)**^a^**	24 (10.9)	0.028
**Included TT**P	84 (94.4)	64 (85.3)	17 (77.3)	11 (78.6)	20 (100.0)	196 (79.1)	
**TP (PoBT/previous)**							
**PoBT-TP**	78 (87.6)**^a^**	49 (65.3)**^ab^**	7 (31.8)**^b^**	7 (50.0)**^ab^**	17 (85.0)**^a^**	158 (71.8)	<0.001
**Previous TP**	11 (12.4)	26 (34.7)	15 (69.2)	7 (50.0)	3 (15.0)	62 (29.2)	
**PoBT (** ***n*** ** = 158)**							
**BT-Naive**	49 (62.8)**^abc^**	42 (85.7)**^c^**	7 (100.0)**^bc^**	4 (57.0)**^ab^**	5 (29.4)**^a^**	107 (67.7)	<0.001
**BT-nonNaive**	29 (37.2)	7 (14.3)	0 (0.0)	3 (43.0)	12 (71.6)	51 (32.3)	

Values are expressed as *n*(%). BT: Biologic therapeutics, PoBT: patients with ongoing BT, TP: Treatment periods, TTP: Total TP, UST: Ustekinumab, ADA: Adalimumab, ETN: Etanercept, IFX: Infliximab, SEC: Secukinumab. Different superscripts in the same row indicates a statistically significant difference among groups.

**Table 5:** Mean and median survival times of total treatment periods of BT (treatment periods of PoBT and previous treatment periods of BT-nonNaive patients).

BT	Mean (95% CI)	Median (95% CI)
**UST (** ***n*** ** = 84)**	14.91(13.32–16.50)	16.00(12.74–19.26)
**ADA (** ***n*** ** = 64)**	13.30(11.03–15.56)	12.00(10.17–13.83)
**ETN (** ***n*** ** = 17)**	11.07(7.61–14.53)	10.00(6.83–13.17)
**INX (** ***n*** ** = 11)**	4.91(3.58–6.24)	6.00(4.26–7.74)
**SEC (** ***n*** ** = 20)**	4.65(3.63–5.67)	4.00(1.81–6.19)
**Total (** ***n*** ** = 196)**	12.39(11.24–13.54)	12.00(10.35–13.65)

BT: Biologic therapeutics. PoBT: Patients with ongoing treatment of BT. TP: Treatment periods. CI: Confidence interval.

Log-rank χ^2^ = 80.688, *p* < 0.001

**Table 6:** Mean and median survival times of PoBT treatment periods

BT	Mean (95% CI)	Median (95% CI)
**UST (** ***n*** ** = 78)**	15.49(13.86–17.12)	16.00(14.27–17.73)
**ADA (** ***n*** ** = 49)**	15.37(12.58–18.16)	16.00(10.89–21.11)
**ETN (** ***n*** ** = 7)**	14.00(9.20–18.80)	-
**INX (** ***n*** ** = 7)**	5.00(3.40–6.60)	4.00(0.80–7.20)
**SEC (** ***n*** ** = 17)**	4.59(3.42–5.75)	4.00(1.98–6.02)
**Total (** ***n*** ** = 158)**	13.61(12.28–14.94)	14.00(11.69–16.31)

BT: Biologic therapeutics. PoBT: Patients with ongoing treatment of BT. TP: Treatment periods. CI: Confidence interval.

Log-rank χ^2^ = 79.915, *p* < 0.001

**Table 7:** Univariate and multiple Cox regression analysis results in identifying the risk factors of ongoing BT survival.

Variables	Univariate		Multiple	
	HR (95% CI)	*p value*	HR (95% CI)	*p value*
**Age**	1.003 (0.988–1.020)	0.676	-	-
**Sex (female/male)**	1.062 (0.744–1.516)	0.741	-	-
**Age of onset (>40 / <=40)**	1.710 (1.057–2.766)	0.029	1.710 (1.057–2.766)	0.029
**Family history of psoriasis**	0.948 (0.634–1.416)	0.794	-	-
**Accompanying disease**	1.038 (0.704–1.532)	0.849	-	-
**Body mass index (kg/m** **^2^** **)**	0.992 (0.961–1.024)	0.624	-	-
**Psoriatic arthritis**	1.188 (0.788–1.789)	0.411	-	-
**Biologic therapeutic naive**	0.756 (0.511–1.118)	0.161	-	-
**Therapies**				
**MTX**	0.90(0.60–0.1.36)	0.621	-	-
**ACN**	1.01(0.69–1.49)	0.942	-	-
**CYC**	1.21(0.84–1.74)	0.307	-	-
**TPC**	2.31(0.32–16.60)	0.404	-	-
**PHT**	1.21(0.84–1.73)	0.303	-	-

HR: Hazard ratio, CI: Confidence interval.

## Footnotes

1 Global report on psoriasis (2014). Website https://apps.who.int/iris/bitstream/handle/10665/204417/9789241565189_eng.pdf;jsessionid=FB89475EFEFF5717AA3092B27F53F385?sequence=1 [6 September 2021].
